# Eosinophils in human oral squamous carcinoma; role of prostaglandin D2

**DOI:** 10.1186/1476-9255-10-4

**Published:** 2013-01-31

**Authors:** Francis Davoine, Adrian Sim, Charlie Tang, Sibina Fisher, Caroline Ethier, Lakshmi Puttagunta, Yingqi Wu, W Tim McGaw, Donald Yu, Lisa Cameron, Darryl J Adamko, Redwan Moqbel

**Affiliations:** 1 Pulmonary Research Group, University of Alberta, 559 Heritage Medical Research Centre, Edmonton, Alberta T6G 2S2, Canada; 2 Campus-Saint-Jean, University of Alberta, 8406 Marie-Anne-Gaboury Street, Edmonton, Alberta T6C 4G9, Canada; 3 Department of Laboratory Medicine and Pathology, University of Alberta, Edmonton, Alberta, Canada; 4 Department of Medical Microbiology and Immunology, University of Alberta, Edmonton, Alberta, Canada; 5 Department of Dentistry, University of Alberta, Edmonton, Alberta, Canada; 6 Department of Paediatrics, University of Saskatchewan, Saskatoon, Saskatchewan, Canada; 7 Department of Immunology, University of Manitoba, Winnipeg, Alberta, Canada

**Keywords:** Eosinophils, Oral squamous carcinoma, Prostaglandin D2, HQL-79, Transmigration

## Abstract

Eosinophils are often predominant inflammatory leukocytes infiltrating oral squamous carcinoma (OSC) sites. Prostaglandins are secreted by oral carcinomas and may be involved in eosinophil infiltration. The objective of this study was to determine the factors contributing to eosinophil migration and potential anti-neoplastic effects on OSC. Eosinophil degranulation was evaluated by measuring release of eosinophil peroxidase (EPO). Eosinophil chemotaxis towards OSC cells was assessed using artificial basement membrane. Eosinophil infiltration was prominent within the tissue surrounding the OSC tumor mass. We observed growth inhibition of the OSC cell line, SCC-9, during co-culture with human eosinophils, *in vitro,* which correlated with EPO activity that possesses growth inhibitory activity. The PGD_2_ synthase inhibitor, HQL-79, abrogated migration towards SCC-9. Our data suggest that OSC-derived PGD_2_ may play an important role via CRTH2 (the PGD_2_ receptor on eosinophils) in eosinophil recruitment and subsequent anti-tumor activity through the action of eosinophil cationic proteins.

## Background

Eosinophils are bone marrow-derived, tissue-dwelling granulocytes found transiently in the blood circulation en route to tissue inflammatory sites. They are prominent cells in allergic inflammation, asthma and parasitic helminth infections [1]. Tumor associated tissue eosinophilia (TATE) has long been recognized as a pathological feature associated with a number of malignant tumor types, including cancer of the mouth, esophagus, larynx, pharynx, breast, lung, intestine and genitourinary tract [2]. Although mononuclear cells, and to a lesser extent neutrophils, are also found in oral cancers, eosinophils when present, form the predominant inflammatory cell population [3-5]. While there is evidence of a positive prognosis associated with eosinophil infiltration of grade II and III tumours [6], in well-differentiated oral cancer of grade III and IV, the presence of TATE indicates a poor prognosis [7]. Regardless, data on the prognostic value of TATE in other cancer types remain inconclusive [2].

OSC are known to express cycloxygenase-2 (COX-2) and to generate PGE_2_[8-11]. In fact, expression of COX-2 and PGE_2_ was thought to be related the proliferation and invasiveness of OSC [12-15]. PGE_2_ does not possess chemotactic activity for eosinophils, but share the same precursor, PGH_2_, with a potent eosinophil chemotactic molecule, PGD_2_[16-18]. In this study we hypothesized that OSC synthesize and release PGD_2_ which in turn is responsible for specific chemotaxis of eosinophils towards OSC.

## Methods

Paraffin embedded tissues of the corresponding sections were sectioned at 4 microns. The sections were then deparaffinized and hydrated in distilled water and stained in a Weigert’s iron hematoxylin and Biebrich scarlet solution (9:1). A differentiation step in 1% acid alcohol was then performed on the sections until the eosinophil granules stained bright red, followed by rinsing in tap water. The sections were then stained in a 0.5% lithium carbonate solution until they turned blue. A final rinsing step was performed before the final slide mounting steps.

### Blood eosinophil isolation

Approval for the study was obtained from the local Ethics Research Board at the Faculty of Medicine and Dentistry (University of Alberta) and all adult subjects gave their informed consent according to the Helsinki protocol. Blood eosinophils from atopic donors were purified as previously described [19]. Briefly, venous blood (100 ml) was collected in tubes with heparin. Red cells were sedimented using Dextran 6% (Sigma-Aldrich Canada Ltd. Oakville, Ontario, Canada). Granulocytes were separated from mononuclear cells by centrifugation on Ficoll Paque. Eosinophils were separated from neutrophils by CD16 immunomagnetic negative selection using a magnetic cell sorter (Miltenyi Biotec GmbH, Bergisch Gladbach, Germany). Purity of eosinophil preparations was always greater than 98%, the contaminating cells being neutrophils and/or lymphocytes.

### Co-culture assay

SCC9 (American Type Culture Collection (ATCC), Manassas, Virginia) were seeded at 5 × 10^4^ cells/ml in 12 well plates 24h prior addition of eosinophils at a range of eosinophil-SCC9 concentrations. Plates were cultured until confluence of SCC9 cells was reached in control wells. Eosinophils were then removed and adherent cells were fixed with 4% paraformaldehyde (Sigma-Aldrich Canada Ltd. Oakville, Ontario, Canada) for 20 minutes and stained with crystal violet 1% (Sigma-Aldrich Canada Ltd. Oakville, Ontario, Canada). After a PBS wash, cells were lysed with 15% acetic acid and absorbance (550 nm) was measured. Absorbance was in the linear range for 2 × 10^2^ to 2 × 10^6^ eosinophils/well.

### Transmigration assay

The transmigration of eosinophils through the basement membrane components was evaluated in 24-well Biocoat Matrigel Invasion Chambers® (Becton Dickinson, Bedford, MA) as previously described [20]. Before the assay, SCC-9 cells were allowed to grow to confluence in the lower chambers, washed and fresh media replaced before the addition of 0.5 × 10^6^ eosinophils in the upper chamber. PGD_2_ synthase inhibitor HQL-79 (Cayman Chemical, Ann Arbor, MI.) (0.1-100 μM) was added to lower chambers to block the production of PGD_2_. HQL-79 is an orally available selective inhibitor of hematopoietic prostaglandin synthase specific with an IC50 of 100 uM for PGD2 but marginally affect the production of other prostanoids [21,22]. The percentage of transmigration was calculated by dividing the number of cells in the lower chamber of the Matrigel Invasion Chamber® by total number of cells in the lower (migrated) and upper chamber (non-migrated). The proportion of cells recovered was always >95% of the number of added cells and viability always above 98% as determined by annexin V/PI assay (n = 3).

### EPO assay

Measurement of EPO release has been previously described [19,23]. Briefly, eosinophil suspensions (5 × 10^5^ cells/ml HBSS + CaCl_2_ 1.6 mM, 0.1% gelatin) were distributed in 96-well plate. Cells were incubated for 30 min at 37°C. Peroxidase substrate solution for the measurement of EPO was prepared by adding 800 μL of 5 mM O-phenylenediamine HCl (OPD) (Sigma-Aldrich Canada Ltd. Oakville, Ontario, Canada) to 4 ml of 1M Tris buffer (pH 8.0) and 1.25 μL of 30% hydrogen peroxide (Sigma). Distilled water was added to a total volume of 10 ml. The OPD solution was added to each well of eosinophils, incubated for 2 minutes before the reaction was stopped with the addition of 4M H_2_SO_4_ (Sigma-Aldrich Canada Ltd. Oakville, Ontario, Canada). Each experiment was done in triplicate and absorbance reading at 490 nm wavelength was done for this colorimetric assay (Softmax, 490 nm wavelength).

### PGD_2_ dosage

Detection of PGD_2_ in media after transmigration was achieved using by EAI according to manufacturer instructions (Prostaglandin D_2_ EIA Kit, Cayman chemical, Ann Arbor, MI).

### Viability assay

The apoptosis/viability assay was performed using the BD Bioscience Annexin-V-A488 detection kit according to manufacturer instructions. Acquisition was performed using BD FACS-CANTO flow cytometer. Viable cells were double negatives for ToPRO-3 and Annexin-V-A488.

### Flow cytometry

Eosinophils were incubated in the presence of anti-CRTH-2-PE (MB16, RatIgG2a, Miltenyi Biotec, Auburn, CA) or matched isotype control antibody. Direct staining was used to detect the presence of specific surface binding with BD FACScanto flow cytometer.

### Statistical methods

All results are expressed as mean ± standard error of mean. Comparison between the groups was made using analysis of variance (ANOVA; Statview 5.0, SAS Institute, Cary, NC). A p-value <0.05 was considered significant.

## Results

### Eosinophil infiltration in oral squamous carcinoma

**Figure 1:**
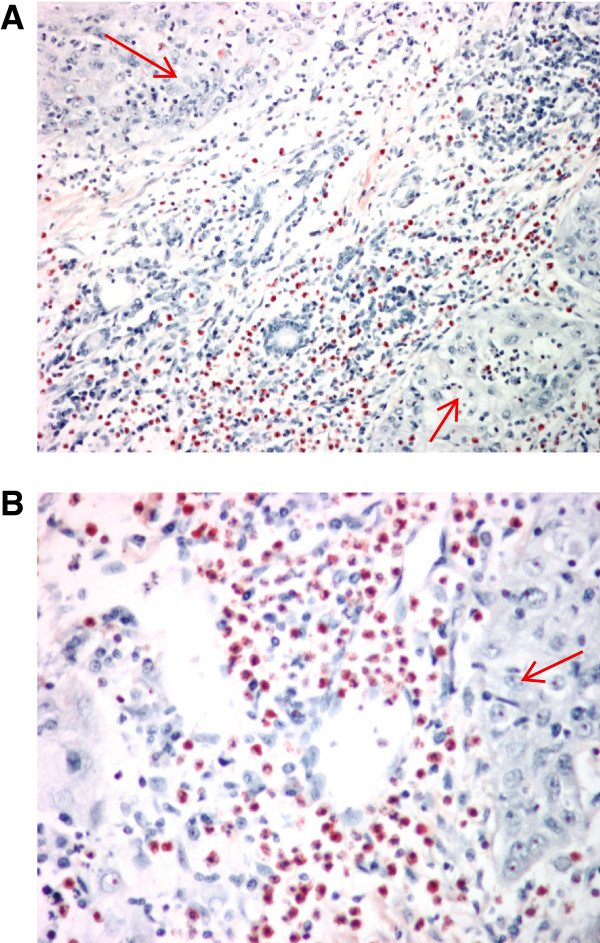
**TATE is observed predominantly around the tumor mass. A**: Luna staining for eosinophils in surgically-resected primary oral squamous cancer. **B**: higher magnification clearly shows that Infiltration of eosinophils is observed around tumor mass. Arrows indicate tumor mass.

Paraffin embedded oral squamous carcinomas specimens were obtained post-surgically from a cohort of 21 subjects. Evidence of eosinophil infiltration was observed around the tumor mass. Figure 1A depicts a specimen with massive eosinophil infiltration in connective tissue. In contrast, very few eosinophils were detected in the tumour cellular compartment (Figure 1B).

### PGD_2_ secretion by OSC elicit eosinophil migration

**Figure 2:**
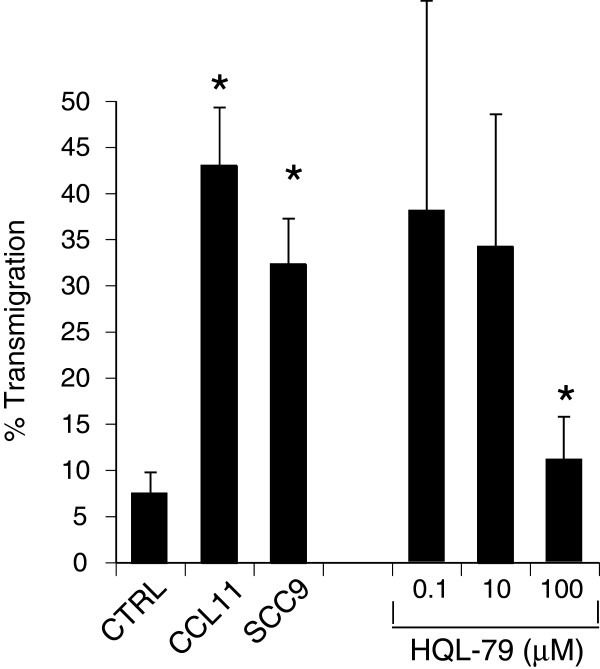
**Eosinophil migrates through artificial basal membrane (Matrigel) toward SCC9.** CCL11 (eotaxin 0.01μm) was used as positive control for transmigration assay. PGD2 synthase inhibitor (HQL-79)-treated SCC9 did not induce eosinophil transmigration (n = 9, Mean ± SEM, p < 0.05).

To examine the capacity of OSC to elicit eosinophil migration we used a migration system that uses an artificial cell basement membrane (Matrigel™) to separate compartment. Eosinophils did not migrate spontaneously through the lower chamber in the absence of SCC9 cells (7 ± 2%, n = 20). In comparison, the confluent monolayer of SCC9 cells induced migration toward OSC through matrigel (Figure 2, 32 ± 5% n = 17, p < 0.05). To determine whether PGD_2_ played a role in this migration, a PGD_2_ synthase inhibitor (HQL-79) was used. Before migration, the OSC monolayer was washed with fresh media to ensure only *de novo* generation of PGD_2_ was affected by the HQL-79 inhibitor. A dose response effect of HQL-79 on SCC9 induced eosinophil migration was observed. At a maximal concentration (100 μM) HQL-79 reduced the migration to a level similar to background spontaneous migration (11 ± 7%, n = 9, p < 0.05). The positive control, CCL-11 (eotaxin-1), induced migration at a level of 43 ± 6% (n = 20, p < 0.05). HQL-79 did not impair CCL-11-induced migration or reduced eosinophil viability as determined by flow cytometry (data not shown). The secretion PGD_2_ was also confirmed by enzyme-linked immunosorbent assay (EIA) in SCC9 confluent wells after transmigration, a mean concentration 1.5 ± 0.4 nM of PGD_2_ was detected in the 4 experiments tested. The addition of HQL-79 (100 μM) reduced the PGD_2_ concentration below detection limit.

### Eosinophils inhibited OSC growth

**Figure 3:**
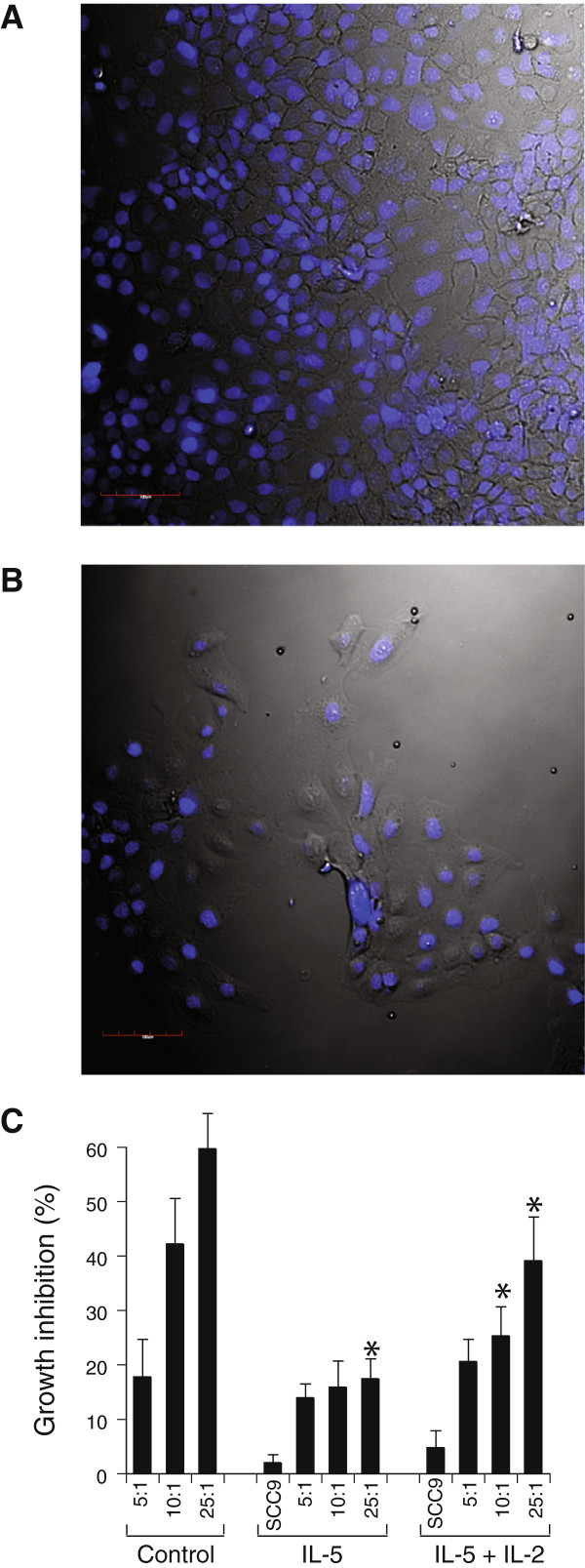
**Eosinophils inhibit the formation of monolayers of the oral cancer cell line, SCC9. A**: Representative confluent monolayer of SCC9, 5 days after culture, Nuclei are stained with DAPI for easier identification of SCC9 after washing out eosinophils (red bar = 100 μm). Eosinophils were removed from wells before staining **B**: Co-culture with eosinophils prevented SCC9 cell growth. **(**25:1 eosinophil-SCC9 ratio in media) **C**: Mean growth inhibition observed in 5 independent experiments with different eosinophil-SCC9 ratio. IL-5 was used to sustain eosinophil viability except in control group. Reference group (0% growth inhibition) represent SCC9 incubated without addition of cytokines or eosinophils that reached confluence (5 days). SCC9 + IL-5 and SCC9 + IL-5 + IL-2 represent control groups for the effect of cytokines without eosinophil co-incubation. All data are represented by Mean ± SEM * p < 0.05.

Figure 3A shows a representative confluent layer of OSC after 5 days of culture. In contrast, adding eosinophils prevented OSC from reaching confluence (Figure 3B). In an attempt to potentiate eosinophil survival and function and potentially decrease tumor growth, we added IL-2 (100 ng/ml), IL5 or a combination to the culture media. Figure 3C depicts the mean results of 5 independent co-culture assays. Maximum growth inhibition (60 ± 7%, n = 5, p < 0.05) was achieved in co-culture with a 25:1 ratio of eosinophil: SCC9. The growth of OSC was unaffected by treatment with IL-5 alone (10 ng/ml; SCC9 + IL-5). In conditions were SCC9 + IL-5 + eosinophils (25:1) were co-incubated inhibition reached only 18 ± 4% (n =5, p < 0.05). The addition of IL-5 and IL-2 increased SCC9 growth inhibition when co-incubated with eosinophils ratio of 10:1 (25 ± 6%, n = 5) and 25:1 (39 ± 8%, n = 5, p < 0.05).

### Growth inhibition of OSC was associated with eosinophil mediator release and eosinophil cytolysis

**Figure 4:**
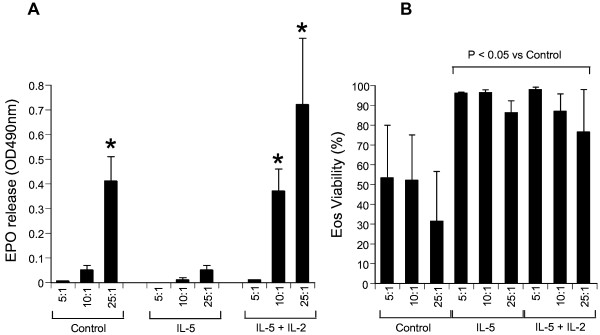
**SCC9 growth inhibition correlate with EPO release and cell viability. A**: IL-2 induced release of Eosinophil peroxidase (EPO) in culture media is associated with inhibition of oral cancer cell proliferation. EPO was measured by a colorimetric assay using the substrate *O*-phenylenediamine dihydrochloride. **B**: Viability of eosinophils in co-culture with oral squamous cancer cells. Viability was measured using flow cytometry by assessing cell integrity using Topro3 DNA dye and absence of apoptosis with Annexin V. All data are represented by Mean ± SEM * p < 0.05 for eosinophil:SCC9 co-culture compared with the respective controls without cytokines.

Eosinophil peroxidase (EPO) is a potent cytotoxic protein stored by eosinophils. We observed two forms of EPO release: first from necrotic non-IL-5-treated eosinophils and those stimulated by IL-2. In contrast, negligible EPO activity was detected in the supernatant of IL-5 treated co-culture of cells associated with the minimum growth inhibition (Figure 4A). Eosinophils viability in co-culture medium was measured immediately after control OSC reached confluence (after 5 days). Eosinophil viability was reduced under medium alone conditions but remained well above 80% in medium containing IL-5 (Figure 4B). In IL-2 + IL-5-treated groups, eosinophil viability remained unchanged indicating that the EPO activity measured was unlikely to be attributable to necrosis. Since IL-5 alone did not induce significant EPO release or OSC growth inhibition, IL-2 plus IL-5 appeared to induce EPO release without exerting an effect on eosinophil viability thereby contributing to OSC growth inhibition. These results indicated that OSC growth inhibition in untreated medium might be a result of eosinophil necrosis, cell membrane disruption and inevitable toxic mediator release by cytolysis. Maximum EPO activity correlated with maximum growth inhibition. These results suggest that cytotoxic mediator release may be a mechanism by which eosinophil inhibit the growth of OSC.

## Discussion

Eosinophil infiltration around tumor nests is a frequent feature of OSC and is accompanied by a mixed lymphocyte response [6]. This infiltration often correlates with deposition of eosinophils cytotoxic proteins with favorable prognosis but the mechanism remains unclear [24]. The *de novo* secretion of PGD_2_ by the OSC cell line, SCC-9, the matrigel transmigration experiments, and inhibition with the PGD_2_ synthase inhibitor, HQL-79, all combine to suggest that PGD_2_ may be an important mediator in tumor-induced recruitment of eosinophils.

Previous studies have reported PGE_2_ secretion by OSC [15,25,26] but there has been no evidence that this prostaglandin exerts chemoattractive activity on eosinophils; however a closely related prostaglandin, PGD_2_, is known for its chemotactic activity on eosinophils [16-18]. In this study we report that eosinophils exhibit potent growth-inhibitory activity against the oral cancer cell line, SCC9 which was associated with eosinophil specific EPO release in culture medium. There is no evidence to date that cytotoxic eosinophil granule deposition plays a role *in vivo* and no data so far exist to support a correlation between granule deposition in OSC and favorable prognosis. In our experiments, we observed that inhibition of OSC growth correlated with detectable cytotoxic granule enzyme EPO activity in culture medium. This association between OSC growth inhibition and eosinophil mediator release was observed regardless of eosinophil viability in the absence of factors that sustain viability (IL-5) thus disrupting eosinophil cell membranes resulting in a non-specific cytolytic release of granular content.

Immunotherapy using IL-2 has been shown to have moderate success against some tumors and is often associated with “unexpected” but significant eosinophilia [27], which resulted in assumptions suggesting that eosinophils possess anti-tumor activity, at least *in vitro*[28]. Indeed, IL-2 is recognized as a potent regulator of eosinophil activation, *in vitro*[29,30]*.* The effects of IL-2 include the release of cytotoxic granules, generation of superoxide radicals [31,32] and production of autocrine IL-2 [29,30]. IL-2-induced TATE (corollary to treatment of renal cancer), close proximity of activated eosinophils with bladder tumor cells and the subsequent deposition of eosinophil cationic granules were shown to be associated with a favourable outcome [33]. In contrast, the presence of IL-2-induced eosinophilia was considered predictive of the failure of therapy in renal cancer [34].

Despite the significant *in vitro* effects we observed with OSC, eosinophils appeared to be mostly recruited around, but not within OSC masses. As well, there was very little evidence of eosinophil granule deposition *ex vivo*. Regardless, basic proteins from eosinophil granules are extremely cytotoxic, thus, small concentration of free exocytosed granules may be sufficient to exert a potent inflammatory/cytotoxic response against tumor cells [35]. Recent studies from our group suggested that cell-free granules from eosinophils can secrete their content *via* direct stimulation of functional cytokine and chemokine membrane receptors for present on the granule membrane, in the absence of an intact cell [36]. In addition to these potential cytotoxic effector activities, eosinophils are also capable of exerting an immunoregulatory role in relation to the tumor environment. Eosinophils secrete a wide range of cytokines chemokines and growth factors [37] and these may further contribute to the biological and immunological role of the eosinophil in OSC.

Finally, the cyclooxygenase-2 (COX-2) inhibitor, NS398, was reported to inhibit OSC proliferation by suppressing PGE_2_ secretion [26]. However, PGE_2_ is not a chemoattractant for eosinophils. In contrast, we show that PGD_2_ is a potent chemoattractant for eosinophils, and may contribute to eosinophilic infiltration via its specific PGD_2_ receptor on eosinophil, CRTH2, which is also a marker of TH-2 subset of helper T-cells [16]. Whether PGD_2_ also enhances the potential of eosinophils to kill target cells by inducing exocytosis and subsequent deposition of cytotoxic granule proteins remains unknown and is the subject of a separate study. Thus, our data suggest that eosinophils may contribute to the inflammatory response observed in OSC and may limit tumor progression.

## Competing interest

The authors declare that they have no competing interests

## Authors’ contribution

FD wrote the manuscript and was primarily responsible for the acquisition of data, analysis and data interpretation. FD and RM also designed the study. AS, CT, SF, YW and CE were also involved in acquisition of data, analysis and interpretation of data. YW contributed to the design of the EPO assay and collection of data. LP, DY and TMcG contributed to the collection and supervision of the pathological specimen collection and review of the scientific content of the manuscript. LC provided important intellectual input specifically regarding PGD2 and CRTH2 on eosinophil migration, and was also involved in the intellectual aspect of the scientific content of the manuscript. RM, DA and DY were co-supervisors of FD, with RM being the principal investigator for this study from conception, design, analysis, and interpretation to writing of the manuscript and final approval of the submitted and revised version of the ms. All authors read and approved the final manuscript.

## References

[B1] Adamko DJ Odemuyiwa SO Vethanayagam D Moqbel R The rise of the phoenix: the expanding role of the eosinophil in health and disease Allergy 2005 60 13 22 10.1111/j.1398-9995.2005.00676.x 15575925

[B2] Samoszuk M Eosinophils and human cancer Histol Histopathol 1997 12 807 812 9225164

[B3] Falconieri G Luna MA Pizzolitto S DeMaglio G Angione V Rocco M Eosinophil-rich squamous carcinoma of the oral cavity: a study of 13 cases and delineation of a possible new microscopic entity Ann Diagn Pathol 2008 12 322 327 10.1016/j.anndiagpath.2008.02.008 18774493

[B4] Alkhabuli JO High AS Significance of eosinophil counting in tumor associated tissue eosinophilia (TATE) Oral Oncol 2006 42 849 850 10.1016/j.oraloncology.2005.11.023 16829160

[B5] Alrawi SJ Tan D Stoler DL Dayton M Anderson GR Mojica P Douglas W Hicks W Jr Rigual N Loree T Tissue eosinophilic infiltration: a useful marker for assessing stromal invasion, survival and locoregional recurrence in head and neck squamous neoplasia Cancer J 2005 11 217 225 10.1097/00130404-200505000-00008 16053665

[B6] Dorta RG Landman G Kowalski LP Lauris JR Latorre MR Oliveira DT Tumour-associated tissue eosinophilia as a prognostic factor in oral squamous cell carcinomas Histopathology 2002 41 152 157 12147093 10.1046/j.1365-2559.2002.01437.x

[B7] Horiuchi K Mishima K Ohsawa M Sugimura M Aozasa K Prognostic factors for well-differentiated squamous cell carcinoma in the oral cavity with emphasis on immunohistochemical evaluation J Surg Oncol 1993 53 92 96 10.1002/jso.2930530209 8501912

[B8] Chan G Boyle JO Yang EK Zhang F Sacks PG Shah JP Edelstein D Soslow RA Koki AT Woerner BM Cyclooxygenase-2 expression is up-regulated in squamous cell carcinoma of the head and neck Cancer Res 1999 59 991 994 10070952

[B9] Mohammed SI Coffman K Glickman NW Hayek MG Waters DJ Schlittler D DeNicola DB Knapp DW Prostaglandin E2 concentrations in naturally occurring canine cancer Prostaglandins Leukot Essent Fatty Acids 2001 64 1 4 10.1054/plef.2000.0231 11161579

[B10] Pannone G Bufo P Caiaffa MF Serpico R Lanza A Lo Muzio L Rubini C Staibano S Petruzzi M De Benedictis M Cyclooxygenase-2 expression in oral squamous cell carcinoma Int J Immunopathol Pharmacol 2004 17 273 282 15461861 10.1177/039463200401700307

[B11] Renkonen J Wolff H Paavonen T Expression of cyclo-oxygenase-2 in human tongue carcinoma and its precursor lesions Virchows Arch 2002 440 594 597 10.1007/s00428-002-0616-y 12070598

[B12] Kinugasa Y Hatori M Ito H Kurihara Y Ito D Nagumo M Inhibition of cyclooxygenase-2 suppresses invasiveness of oral squamous cell carcinoma cell lines via down-regulation of matrix metalloproteinase-2 and CD44 Clin Exp Metastasis 2004 21 737 745 16035618 10.1007/s10585-005-1190-x

[B13] Mestre JR Chan G Zhang F Yang EK Sacks PG Boyle JO Shah JP Edelstein D Subbaramaiah K Dannenberg AJ Inhibition of cyclooxygenase-2 expression. An approach to preventing head and neck cancer Ann N Y Acad Sci 1999 889 62 71 10.1111/j.1749-6632.1999.tb08724.x 10668483

[B14] Minter HA Eveson JW Huntley S Elder DJ Hague A The cyclooxygenase 2-selective inhibitor NS398 inhibits proliferation of oral carcinoma cell lines by mechanisms dependent and independent of reduced prostaglandin E2 synthesis Clin Cancer Res 2003 9 1885 1897 12738747

[B15] Sumitani K Kamijo R Toyoshima T Nakanishi Y Takizawa K Hatori M Nagumo M Specific inhibition of cyclooxygenase-2 results in inhibition of proliferation of oral cancer cell lines via suppression of prostaglandin E2 production J Oral Pathol Med 2001 30 41 47 10.1034/j.1600-0714.2001.300107.x 11140899

[B16] Fujishima H Fukagawa K Okada N Takano Y Tsubota K Hirai H Nagata K Matsumoto K Saito H Prostaglandin D2 induces chemotaxis in eosinophils via its receptor CRTH2 and eosinophils may cause severe ocular inflammation in patients with allergic conjunctivitis Cornea 2005 24 S66 S70 10.1097/01.ico.0000178733.42921.4c 16227827

[B17] Hirai H Tanaka K Yoshie O Ogawa K Kenmotsu K Takamori Y Ichimasa M Sugamura K Nakamura M Takano S Nagata K Prostaglandin D2 selectively induces chemotaxis in T helper type 2 cells, eosinophils, and basophils via seven-transmembrane receptor CRTH2 J Exp Med 2001 193 255 261 10.1084/jem.193.2.255 11208866 PMC2193345

[B18] Monneret G Gravel S Diamond M Rokach J Powell WS Prostaglandin D2 is a potent chemoattractant for human eosinophils that acts via a novel DP receptor Blood 2001 98 1942 1948 10.1182/blood.V98.6.1942 11535533

[B19] Davoine F Ferland C Chakir J Lee JE Adamko DJ Moqbel R Laviolette M Interleukin-12 inhibits eosinophil degranulation and migration but does not promote eosinophil apoptosis Int Arch Allergy Immunol 2006 140 277 284 10.1159/000093705 16735797

[B20] Ferland C Guilbert M Davoine F Flamand N Chakir J Laviolette M Eotaxin promotes eosinophil transmigration via the activation of the plasminogen-plasmin system J Leukoc Biol 2001 69 772 778 11358986

[B21] Aritake K Kado Y Inoue T Miyano M Urade Y Structural and functional characterization of HQL-79, an orally selective inhibitor of human hematopoietic prostaglandin D synthase J Biol Chem 2006 281 15277 15286 10.1074/jbc.M506431200 16547010

[B22] Schuligoi R Sedej M Waldhoer M Vukoja A Sturm EM Lippe IT Peskar BA Heinemann A Prostaglandin H2 induces the migration of human eosinophils through the chemoattractant receptor homologous molecule of Th2 cells, CRTH2 J Leukoc Biol 2009 85 136 145 18835884 10.1189/jlb.0608387

[B23] Adamko DJ Wu Y Gleich GJ Lacy P Moqbel R The induction of eosinophil peroxidase release: improved methods of measurement and stimulation J Immunol Methods 2004 291 101 108 10.1016/j.jim.2004.05.003 15345309

[B24] Pereira MC Oliveira DT Kowalski LP The role of eosinophils and eosinophil cationic protein in oral cancer: a review Arch Oral Biol 2011 56 353 358 10.1016/j.archoralbio.2010.10.015 21112047

[B25] Hoshikawa H Goto R Mori T Mitani T Mori N Expression of prostaglandin E2 receptors in oral squamous cell carcinomas and growth inhibitory effects of an EP3 selective antagonist, ONO-AE3-240 Int J Oncol 2009 34 847 852 19212690 10.3892/ijo_00000211

[B26] Husvik C Khuu C Bryne M Halstensen TS PGE2 production in oral cancer cell lines is COX-2-dependent J Dent Res 2009 88 164 169 10.1177/0022034508329519 19278989

[B27] van Haelst Pisani C Kovach JS Kita H Leiferman KM Gleich GJ Silver JE Dennin R Abrams JS Administration of interleukin-2 (IL-2) results in increased plasma concentrations of IL-5 and eosinophilia in patients with cancer Blood 1991 78 1538 1544 1884020

[B28] Rivoltini L Viggiano V Spinazze S Santoro A Colombo MP Takatsu K Parmiani G In vitro anti-tumor activity of eosinophils from cancer patients treated with subcutaneous administration of interleukin 2. Role of interleukin 5 Int J Cancer 1993 54 8 15 10.1002/ijc.2910540103 8386711

[B29] Bosse M Audette M Ferland C Pelletier G Chu HW Dakhama A Lavigne S Boulet LP Laviolette M Gene expression of interleukin-2 in purified human peripheral blood eosinophils Immunology 1996 87 149 154 8666427 PMC1383981

[B30] Levi-Schaffer F Barkans J Newman TM Ying S Wakelin M Hohenstein R Barak V Lacy P Kay AB Moqbel R Identification of interleukin-2 in human peripheral blood eosinophils Immunology 1996 87 155 161 8666429 PMC1383982

[B31] Conesa A Tassinari P Aldrey O Taylor P Bianco NE De Sanctis JB Interleukin-2 induces peroxide production by primed normodense eosinophils of patients with asthma Allergy Asthma Proc 2003 24 27 33 12635575

[B32] Valerius T Repp R Kalden JR Platzer E Effects of IFN on human eosinophils in comparison with other cytokines. A novel class of eosinophil activators with delayed onset of action J Immunol 1990 145 2950 2958 1698867

[B33] Huland E Huland H Tumor-associated eosinophilia in interleukin-2-treated patients: evidence of toxic eosinophil degranulation on bladder cancer cells J Cancer Res Clin Oncol 1992 118 463 467 10.1007/BF01629431 1618895 PMC12201474

[B34] Moroni M Porta C De Amici M Quaglini S Cattabiani MA Buzio C Eosinophils and C4 predict clinical failure of combination immunotherapy with very low dose subcutaneous interleukin-2 and interferon in renal cell carcinoma patients Haematologica 2000 85 298 303 10702820

[B35] Kubo H Loegering DA Adolphson CR Gleich GJ Cytotoxic properties of eosinophil granule major basic protein for tumor cells Int Arch Allergy Immunol 1999 118 426 428 10.1159/000024154 10224465

[B36] Neves JS Perez SA Spencer LA Melo RC Reynolds L Ghiran I Mahmudi-Azer S Odemuyiwa SO Dvorak AM Moqbel R Weller PF Eosinophil granules function extracellularly as receptor-mediated secretory organelles Proc Natl Acad Sci U S A 2008 105 18478 18483 10.1073/pnas.0804547105 19017810 PMC2587599

[B37] Hogan SP Rosenberg HF Moqbel R Phipps S Foster PS Lacy P Kay AB Rothenberg ME Eosinophils: biological properties and role in health and disease Clin Exp Allergy 2008 38 709 750 10.1111/j.1365-2222.2008.02958.x 18384431

